# Perceived Benefits Matter the Most in COVID-19 Preventive Behaviors: Empirical Evidence from Okara District, Pakistan

**DOI:** 10.3390/ijerph18136772

**Published:** 2021-06-24

**Authors:** Gulzar H. Shah, Ansar Ali Faraz, Hina Khan, Kristie C. Waterfield

**Affiliations:** 1 Department of Health Policy and Community Health, Jiann-Ping Hsu College of Public Health, Georgia Southern University, Statesboro, GA 30460, USA; kwaterfield@georgiasouthern.edu; 2 Department of Statistics, Government College University, Lahore 54000, Punjab, Pakistan; ansar.ali@gcu.edu.pk (A.A.F.); hinakhan@gcu.edu.pk (H.K.)

**Keywords:** COVID-19 preventative behaviors, perceived susceptibility, cues to action, health belief model, health education, Okara, Pakistan

## Abstract

The 2019 coronavirus disease (COVID-19), caused by the SARS-CoV-2 virus has affected the social determinants of health, worsening health inequities and deteriorating healthcare capacities around the globe. The objective of this study is to investigate the COVID-19 prevention behaviors within the framework of the Health Belief Model in the city of Depalpur in the Okara District of Pakistan in May 2020. Using an observational, cross-sectional, and quantitative study design, a face-to-face field survey was conducted during the epidemic of COVID-19 in district Okara, Pakistan. A sample of 500 adults was selected from the city of Depalpur the in Okara district of Pakistan, using a two-stage sampling design with cluster sampling in stage one and systematic random sampling at stage two. A COVID-19 prevention behavior scale was computed based on twelve dichotomous items. Descriptive statistics, analysis of variance (ANOVA), and negative binomial regression analyses were performed. The most common prevention behavior among study participants was avoiding going for walks in the parks (81.0%), followed by not leaving home during the lockdown (72.6%), and washing hands every day with soap and water for 20 s after going out of their home (64.0%). Fewer people exhibited prevention behaviors such as social distancing (e.g., staying at least six feet away from other people) which in the EU was recommended to be a minimum of 1.5–2 m (44.4%) and following all of the basic protective measures (e.g., hand washing, use of a face covering in public, social distancing) in order to protect against COVID-19 (33.0%). The results from the negative binomial regression analysis showed that after controlling for the other HBM constructs and sociodemographic factors, only the perceived benefits of preventative actions showed significant association with the prevention behavior scale (IRR, 1.16; CI, 1.061–1.276; *p* < 0.001). The study findings show that public health interventions attempting to control the spread of COVID-19 in Pakistan may want to affect a change in people’s perceived benefits of preventative actions through mass awareness-raising campaigns.

## 1. Introduction

The SARS-CoV-2 virus, which is the cause of the 2019 coronavirus disease (COVID-19) was first reported in late December 2019 in Wuhan in the Hubei province of China [1,2,3]. COVID-19 quickly spread globally and was declared a global pandemic by the World Health Organization (WHO) [4]. This viral disease outbreak overwhelmed global healthcare systems and adversely impacted the routine care for many chronic and ambulatory care-sensitive conditions such as cancer, diabetes, and asthma. It particularly affected resource-poor countries [2,5,6,7,8,9]. The intensity of the dreadful effects of coronavirus was faced by both developed and developing countries. However, in countries with weak healthcare system infrastructures, the effects appeared to be intensified [10,11]. In Pakistan, the first two positive cases of COVID-19 were reported on 26 February 2020. According to documentation, both patients had a history of recent travel to Iran [12]. As of 30 March 2021, a total number of 663,200 cases have been reported in Pakistan, a country with a population of 220.9 million people in 2020 [13], with 14,356 deaths attributable to COVID-19, which many believe is a serious under-estimation due to the severe social stigma associated with this disease [14,15,16]. 

To curb the spread of COVID-19 in Pakistan, protective measures such as social distancing, frequent hand-washing, and the use of face coverings in public were put into place and are still considered essential, given that vaccine distribution and acceptance has been challenging in subgroups with a distrust of science and western medicine [17]. To complicate the matter, global health inequities in affordability and the timing of COVID-19 vaccine sales to low-income countries (in comparison with high-income countries) still leaves preventive measures such as social distancing and face coverings as the only means to control the spread of SARS-CoV-2 [18]. A lack of trust in scientific facts, in favor of here-say and conspiracy theories, can be challenging to science-based interventions concerning COVID-19 [19]. A distrust in science may lead to an inability to learn or believe even the simplest facts about COVID-19. 

In their efforts to overcome and control the spread of SARS-CoV-2, several timely measures were taken by the Pakistan government. These included the first National Coordination Committee (NCC) for COVID-19, which was established to assist with disease surveillance and data-driven decision-making. On 1 May 2020, Pakistan decided to completely close its western border with Afghanistan and Iran (reopened in August 2020). Additionally, all public gatherings and large meetings (≥250 persons or >50% of location capacity) were banned with immediate effect, and all public and private educational institutions (schools, colleges, universities), wedding halls, and cinemas were closed across the country (re-openings began in January 2021) [20,21]. A countrywide face-covering mandate took effect in Pakistan on 31 May 2020. This mandate, along with social distancing, is still in place [22]. Unfortunately, closures of businesses and a rise in unemployment has caused a negative economic impact on the Pakistani economy [23]. 

In Pakistan and around the globe, pandemic fatigue coupled with low health literacy has created a public health crisis that necessitates the need for broader compliance with local and global guidelines for stopping the spread of SARS-CoV-2 [19]. For public health practitioners to overcome this disease, it is necessary to understand what motivates a person’s compliance with preventive measures [24,25]. To generate the practice-relevant evidence, the present study aimed to analyze the correlates of prevention behaviors concerning COVID-19 by using the Health Belief Model (HBM). HBM is one of the models widely used to convey beliefs that can be effective in shaping health protection and health promotion behaviors, including those concerning COVID-19 [26,27,28]. According to HBM, behavioral beliefs and modifying factors can be effective in shaping behavior, especially when someone is susceptible to the disease (perceived susceptibility), they aware of the threat of the disease to their health (perceived severity), and they also know the benefits of protective measures (perceived benefits) rather than their barriers (perceived barriers) [24]. By using this model, we aimed to determine which domains of the Health Belief Model are associated with COVID-19 prevention behaviors in Pakistan.

## 2. Materials and Methods

### 2.1. Study Design

This study used an observational, cross-sectional, and quantitative research design based on primary data collected through a face-to-face survey of the adult population in the city of Depalpur in the Okara district of Pakistan. The population of the city of Depalpur was 74,640 in 2020 [29]. The survey was based on a multi-stage sampling design, with cluster sampling in stage one, each comprising a city block. At the first stage, a total of 30 clusters were included in the sample. The systemic sampling design was used at the second stage of sample selection, with a random start, to sample 17 households per cluster from the clusters sampled at stage one, wherein at least one member of the household was above 19 years of age and was willing to participate in the survey [30]. After random selection of the first household, the subsequent 16 households in each cluster were selected using systematic sampling with equal intervals. When 17 participants (one per household) who agreed to participate in the survey were recruited in a cluster, we terminated the sample selection and moved to the next cluster, finally selecting a sample of 510 participants. 

### 2.2. The Survey Instrument and Data Collection

A structured questionnaire (Figure S1) was constructed by the authors based on the grey literature on COVID-19 attitudes, perceptions, and preventive behaviors recommended by the WHO and other public health organizations [31,32,33]. For the socio-demographic and household characteristics, the authors developed questions suitable for the local context. The questionnaire was translated into Pakistan’s national language, Urdu, and duly pretested and updated before using it for data collection (Figure S1). For pretesting, a convenience sample of 30 participants was recruited from the community in the actual study setting. The participants were asked to complete the questionnaire in a face-to-face setting. After each questionnaire was completed, the participants were asked about their understanding of the questions to assess their face validity. They were also asked which questions they found unclear and why. Based on information from pretesting, the questionnaire was updated. Three interviewers that were trained in face-to-face survey methodology conducted the interviews during the partial lockdown of Pakistan from 17 to 25 May 2020 using the questionnaire. Since the survey participants volunteered to participate at the sampling stage, a total of 500 adults finally participated in the survey with a response rate of 98%.

### 2.3. Measures

The survey items/questions were mapped to constructs of the health belief model, through research team discussion and consensus on mapping the items that could be assigned to more than one of the HBM constructs. As a result of this process, all survey questions about COVID-19-related awareness, knowledge, attitudes, motivations, and behaviors were grouped into HBM contracts (shown in Table 1).

The dependent variable, COVID-19 preventive behaviors/actions, was a scale that consisted of the sum of 12 items, each coded as yes = 1 and no/don’t know = 0. The other constructs of the Health Belief Model, computed the same way as the dependent variable scale, served as the independent variables which included: (a) perceived susceptibility to disease (3 items); perceived severity of disease (3 items); perceived benefits of preventative action (3 items); perceived barriers of preventative action (2 items); and cues to action (2 items). None of the survey items mapped well to the HBM construct perceived self-efficacy. The sociodemographic variable constituted the construct “modifying factors,” which included gender, age, marital status, level of education, occupation, monthly income, and medical history (see supplemental digital Table S1). 

### 2.4. Statistical Methods

Descriptive statistics were used to describe the sample characteristics. To assess the association of individual items in the independent variable scales and demographics variables with COVID-19 preventive behaviors/actions, we computed analysis of variance (ANOVA) with COVID-19 preventive behaviors/actions scale as the dependent variable (mean scale score, 95% confidence interval, and trend *p* values are reported in Table S1 and Table 2). To assess which constructs of HBM were associated with the COVID-19 preventive behaviors/actions score (i.e., a count variable) after controlling for other variables in the multivariable model, we performed the negative binomial regression. We ruled out the use of Poisson Regression because the dependent variable showed overdispersion (Mean = 5.83; variance = 6.49), which violated the assumption of Poisson Regression. All analyses were conducted using IBM SPSS Statistics Version 25.0 [34]. 

### 2.5. Human Subject Protection

This study protocol and materials were reviewed and approved by the Research and Ethics Committee of the Government College University [Protocol number GCU-IIB-380]. Consent was obtained from all participants with a clear declaration that the survey participation was voluntary.

## 3. Results

### 3.1. Demographic Information

Table 1 shows the descriptive statistics for demographic variables. Of the 500 survey participants (one member of each household) 74.0% were male and 26.0% were female. Only 46% had at least a 10th-grade education (high school in Pakistan), whereas 30.6% had no formal education. The majority (66.2%) were married, and the remaining 33.8% were single, separated, or divorced. Houses were generally crowded as 72.2% had five or more household members. The majority had a household monthly income less than Rs 30,000 (the equivalent of United States $191). Sixty-five percent owned their homes and 51.6% were living within a joint family system. 

### 3.2. Scale for Overall Prevention Behaviors Score

Descriptive statistics for items in the Health Belief Model, presented in Table 2, show that the most common prevention behavior shown by 81.0% of all of the survey participants was avoiding going for walks in the parks, followed by not leaving home during the lockdown (72.6%), and washing hands every day with soap and water for 20 s after going out of their home (64.0%). Avoiding any non-essential travel, and avoiding public transportation were the next most common actions, reported by 63.8%, and 63.2% of study participants, respectively. Staying six feet away from the other people (44.4%) and following all basic protective measures (e.g., hand washing, the use of a face covering in public, social distancing) to protect yourself against COVID-19 (33.0%) were other notable behaviors. Descriptive statistics for additional HBM items are also presented in Table 2.

**Figure 1:**
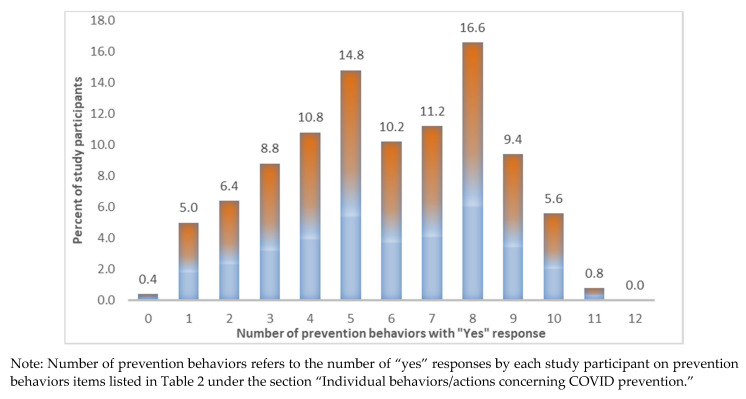
Percent of Study Participants by Number of Prevention Behaviors Concerning COVID-19 (with potential range from 0 to 12).

Figure 1 shows the frequency of count variables of the overall prevention behavior scale. The distribution of COVID-19 preventive behaviors/actions score of the participants towards COVID-19 resembles the normal distribution. One in three or 32.4% of participants indicated performing eight or more preventive actions. A roughly equal proportion (31.4%) performed four or fewer prevention activities. The remaining 36.2% reported between five and seven COVID-19-related preventive actions.

Table 3 shows the results of the ANOVA, comparing means of preventive behaviors/actions score by COVID-19-related statements representing constructs of the HBM. The mean prevention behavior scores for those answering “yes” were significantly different (*p* < 0.05) than those answering “no” for the individual items/statements representing HBM constructs. These constructs included perceived susceptibility to disease, perceived severity of the disease, perceived benefits of preventative action, perceived barriers of preventative action, and cues to action. Comparisons of means for pension behavior score by socio-demographic characteristics are available in Table S1 (supplemental digital content). The differences in prevention behavior scores were noteworthy by the number of people in the household, with smaller size households having significantly higher scores. The mean score for persons with <5 members in the household was 7.74, whereas for those with ≥10 members the mean prevention score was 3.37. Higher prevention behavior scores were observed for individuals with higher education, those younger than 34 years, single/divorced rather than married, and those who owned their homes.

### 3.3. Negative Binomial Regression of COVID-19 Prevention Behaviors

The results from the negative binomial regression analysis (Table 4) showed that after controlling for the other HBM constructs and sociodemographic factors (also referred to as modifying factors in the HBM), only one construct predicted the prevention behavior. The “perceived benefits of preventative actions” score was positively associated with the dependent variable, the individual behaviors/actions concerning COVID prevention (incidence rate ratio, (IRR), 1.16; confidence interval, CI, 1.061–1.276; *p* < 0.001). The IRR 1.16 indicates the estimated ratio of change in prevention behavior score, given a one unit increase in the perceived benefits of prevention score after controlling for all other variables in the model. The other constructs of the HBM, including perceived susceptibility to disease, perceived severity of the disease, perceived benefits of preventative actions, perceived barriers of preventative action, and cues to action had no significant association at *p*≤ 0.05 with the individual behaviors/actions concerning COVID-19 prevention.

## 4. Discussion

Pakistan, like many other countries around the globe, continues to face serious life-threatening effects due to the COVID-19 pandemic, including the emergence of the 3rd wave of COVID-19 cases in late March 2021 [35]. Like many low and middle-income countries (LMICs), Pakistan faces a double jeopardy of COVID-19 vaccine resistance by masses as well as the nonavailability of vaccines. By mid-March 2021, only one million doses of vaccines had been obtained by the Government of Pakistan, with a plan to receive a donation of 10 million doses through Covax, a program co-led by the World Health Organization (WHO) for a country with a population of roughly 221 million [36,37]. With the preventive behaviors being the primary factors that assist with curbing the spread of COVID-19, this study examined the factors associated with COVID-19 prevention behavior using the Health Belief Model. The results of this study revealed that despite the survey being administered during the early months of the pandemic, the overall scores regarding COVID-19, including the perceived susceptibility and perceived severity as well as the perceived benefits and barriers of preventive measures, were high. However, despite apparently adequate levels of knowledge concerning COVID-19 and the necessary preventative measures, the cues to action and the practice of all necessary individual protective behaviors remained relatively low. For instance, social distancing was practiced by 44.4%, and basic protective measures such as handwashing, the use of a face covering in public, etc. to protect against COVID-19 were practiced by 33.0%. Only 16% showed nine or more of the twelve protective behaviors.

### Perceived Benefits Matter the Most

One of our central findings is that among all constructs of the HBM, Pakistani people in Depalpur are moved by the perceived benefits of COVID-19 prevention to comply with prevention guidelines issued by the WHO and the Ministry of Health in Pakistan. When compared to recent studies, the cues to action for the study participants could have been influenced by misinformation regarding the causes and treatment of the SARS-CoV-2 virus, distrust in local government and media figures, misplaced assurance in the effectiveness of nonmedical treatments, and conspiracy narratives related to religious beliefs concerning a viral disease like COVID-19 [19,38]. A recent study in Macao, China, found that perceived susceptibility was the motivation for the participants to comply with prevention guidelines [39].

The other constructs of the HBM, such as perceived susceptibility to getting infected, perceived severity of COVID-19 disease, perceived barriers of preventative action, or cues to action did not seem to alter people’s prevention behaviors. The lack of response to threats and vulnerability may be attributable to a general culture of external locus of control (i.e., that what happens is often out of people’s control) since external threats are numerous and often unavoidable. The general public attitude amounts to “how worse can it get anyway”, given the chronic economic and socio-cultural challenges such as high unemployment and underemployment and extreme social and health inequities.

Perceived benefits in the HBM model refer to an individual’s assessment of the value or efficacy of engaging in a health-promoting behavior to decrease the risk of disease (in this case, COVID-19). The perceived barriers refer to an individual’s feelings concerning the obstacles that may impede their behavior change, which means that when an individual believes a particular action will increase COVID-19 susceptibility or its seriousness, they are thus less likely to engage in preventive behavior [38,39]. Given our findings of primary drivers of behavior change, it is expected that among people of Pakistan, the perceived benefits of any COVID-19 interventions will outweigh the perceived barriers [40,41]. In this study, the perceived barriers were mainly regarding the ability to adhere to recommended social distancing and self-isolation.

In summary, while a range of health behaviors can be explained using HBM, with the exception of benefits, other constructs of HBM did not explain the study participants’ health behaviors in Pakistan. In general, when considering individual behavioral change, poor knowledge and risk perceptions of the disease, illness, or situation are usually considered the main barriers to the change. While the ways in which people perceive risk does not necessarily correlate with the actual risk, their risk perception has been shown to influence their decisions to engage in individual protective behaviors [41]. According to the HBM, an increase in perceived threat (a combination of perceived severity and perceived susceptibility) to a particular health problem would increase engagement in behaviors to reduce their risk of developing the health problem [42,43]. Thus, HBM predicts that individuals who believe they are at low risk of developing an illness are more likely to engage in unhealthy, or risky, behaviors, and those that perceive a higher threat have a higher likelihood of engagement in health-promoting behaviors. In this study, the researchers found that a majority of the study participants believed that there was no cure for COVID-19 (79.4%) and doubted the Pakistani government’s ability to provide proper care for those affected by the virus (68.8%). These beliefs would imply a high perceived severity of the disease and, when combined with high perceived susceptibility, would result in an overall high perceived threat of the virus among the participants.

While the cues to actions were low, the majority of the survey participants was practicing several of the protective measures such as avoiding visiting any crowded place or any social gathering, non-essential travel, and using public transportation. However, many of the participants were not following the basic protective measures (e.g., hand washing, use of face-covering in public, social distancing) when they left their homes. The lack of performing these behaviors could have been due to a number of issues such as the inability to afford soap, hand sanitizer, and/or gloves, as well as a low health literacy. The practicing of protective behaviors was the highest among those between the ages 20–34 years, females, those with at least a high school education, those in homes with less than five people, and single or separated/divorced individuals.

This study’s findings should be interpreted in the context of its several limitations. First, the study is quantitative and cross-sectional. Therefore, many questions could not be answered such as why perceived benefits and not the other constructs of HBM significantly shaped our study participants’ prevention behaviors. Secondly, the self-reported data may have suffered some social desirability bias, particularly because the surveys were conducted in a face-to-face setting. Third, the sample size (*n* = 500) and the study setting may pose limitations on the generalizability of the findings to other parts of the country. Also, we did not conduct the power test to determine the size of the sample, but instead relied on a reasonable sample size estimate. Fourth, the mapping to the HBM model was done after the data collection. It would have been better to do such mapping prior to data collection. Finally, the survey was conducted only three to four months into the COVID-19 pandemic, and some ground realities may have changed later on during the pandemic. Regardless of these limitations, this is an exploratory study that could help the Pakistan government understand the knowledge, attitudes, and practices of the general public concerning COVID-19. Also, the study findings have important implications for COVID-19 vaccine acceptance.

## 5. Conclusions

This study is the first to highlight the knowledge and behavior of the people of the Okara district in Pakistan towards COVID-19. The study showed that while the knowledge and the perceived threat of COVID-19 were high among the citizens of Depalpur City, Pakistan, overall the attitudes and prevention practices of the participants were lower than expected including hand washing, the use of the face-covering in public, and social distancing. While the availability of the SARS-CoV-02 vaccine is extremely limited at this time, our study findings will provide important guidelines to assist in the continued slowing of the spread of COVID-19 and in finding ways for public health professionals to overcome pandemic fatigue.

Our study implies that the government public health agencies in Pakistan should not only do more awareness-raising and health education concerning prevention practices, but that they should also continue to try to galvanize compliance to prevention guidelines by explaining the benefits of compliance with the prevention guidelines issued by the World Health Organization, Pakistan Government, and public health agencies. Although the vaccine is not broadly available currently, in anticipation of its broader availability awareness-raising is imperative about the benefits of the SARS-CoV-2 vaccine for its acceptance. To counter vaccine resistance, public health agencies may also offer benefits such as free vaccination and paid leaves for government workers on days of vaccine appointments. Educational sessions may be organized through civil society organizations in order to increase the overall health literacy of people and, more specifically, to counter the false rumors and conspiracy theories about COVID-19 prevention, management of symptoms, and benefits and side-effects of the COVID-19 vaccine.

## Figures and Tables

**Table 1:** Descriptive Statistics for Socio-Demographic Characteristics of Study Participants, Depalpur City, Pakistan, 2020 (N = 500).

Variable Names	Variable Attributes	Frequency	Percentage
Gender			
	Male	370	74
	Female	130	26
Education			
	No Formal Education	153	30.6
	Less than High School	117	23.4
	High School Diploma	41	8.2
	Intermediate	78	15.6
	Graduation	68	13.6
	Post-Graduation	43	8.6
Age			
	Under 20	78	15.6
	20–34 years	139	27.8
	35–49 years	172	34.4
	50 years and above	111	22.2
Marital Status			
	Single or Separated/Divorced	169	33.8
	Married	331	66.2
Number of Household members			
	Less than five	139	27.8
	Five to Seven	264	52.8
	Eight to Ten	78	15.6
	More than Ten	19	3.8
Monthly Household Income (in Rupees)			
	Less than 30,000 [<$191.45] *	273	54.6
	30,000 to 60,000 [$191.45 to $382.90] *	140	28
	60,000 to 99,000 [$382.90 to $631.78] *	61	12.2
		26	5.2
Medical History			
	No	352	70.4
	Yes	148	29.6
House Ownership			
	No	175	35
	Yes	325	65
Living with Joint Family System			
	No	242	48.4
	Yes	258	51.6

* The equivalent in US Dollars. Abbreviations: N, number of study participants.

**Table 2:** Descriptive Statistics for Items, by the Health Belief Model Constructs, Depalpur City, Pakistan, 2020 [N = 500].

Variable Names	Percent “Yes”
Perceived Susceptibility to Disease	
Are you aware of the common sign and symptoms of COVID-19?	60.2
Could people be unintentionally spreading the COVID-19 virus by touching their cell phones?	29.8
Do you think going to school, hospitals or any institution is safe for you?	32.0
Perceived Severity of Disease	
Is COVID-19 not curable in Pakistan?	79.4
Is Pakistan prepared to provide proper care to people affected by COVID-19 epidemic?	31.2
Has COVID-19 lockdown helped Pakistan prevent its spread?	53.0
Perceived Benefits of Preventative Action	
Do you think social distancing is effective in keeping you safe from COVID-19?	52.0
Do you think that the social distancing slows the rate of COVID-19?	53.2
Do you think that schools should resume quickly after the lockdown period with proper emphasis on social distancing, following the Covid-19 pandemic?	54.0
Perceived Barriers of Preventative Action	
Do you believe COVID-19 related self-isolation and social distancing affect the human body or human mind?	66.6
In your opinion, is your life/family affected negatively by social distancing?	61.6
Cues to Action	
Have you ever been tested for COVID-19?	2.6
Are most of your friends practicing the social distancing?	31.4
Individual behaviors/actions concerning COVID prevention	
Are you avoiding going for walks in the parks?	81.0
Do you not leave your home during the lockdown?	72.6
Are you washing your hands everyday with soap and water for 20 s after you go out of your home?	64.0
Are you avoiding any non-essential travel?	63.8
Are you avoiding using public transportation (except essential service workers)?	63.2
Are you avoiding all social gatherings (large and small)?	61.5
Are you avoiding going to the grocery store or pharmacy?	51.0
Are you staying/working at home rather than going to work or school?	44.6
Are you staying six feet away from the other people?	44.4
Are you self-quarantining if you have the virus or believe you have the virus?	34.8
Are you following basic protective measures (e.g., hand washing, use of mask in public, social distancing) to protect yourself against the COVID-19?	33.0
Are you wearing gloves all the time you go out of your home?	14.4

NOTE: there were no questions in the survey to reflect “Perceived Efficacy”.

**Table 3:** Analysis of variance for comparison of mean number of COVID-19 prevention behavior * by participants’ beliefs and perceptions about COVID-19, Depalpur City, Pakistan, 2020.

COVID-19 Beliefs and Perceptions	Attribute	Mean *	95% C.I.		*p*
			LL	UL	
Do you believe COVID-19 related self-isolation and social distancing affect the human body or human mind?	No/unknown	5.08	4.74	5.43	**<0.001**
	Yes	6.21	6.93	6.49	
Do you think that the social distancing slows the rate of COVID-19?	No/unknown	4.59	4.32	4.85	**<0.001**
	Yes	6.93	6.64	7.22	
In your opinion, is your life/family affected negatively by social distancing?	No/unknown	6.22	5.84	6.6	**0.01**
	Yes	5.59	5.31	5.86	
Has COVID-19 lockdown helped Pakistan prevent its spread?	No/unknown	4.70	4.42	4.98	**<0.001**
	Yes	6.84	5.54	7.13	
Do you think that schools should resume quickly after the lockdown period with proper emphasis on social distancing, following the Covid-19 pandemic?	No/unknown	5.47	5.16	5.78	**0.003**
	Yes	6.14	5.83	6.45	
Do you think going to school, hospitals, or any institution is safe for you?	No/unknown	7.24	6.91	7.56	**<0.001**
	Yes	5.17	4.91	5.44	
Do you think social distancing is effective in keeping you safe from COVID-19?	No/unknown	5.01	4.72	5.3	**<0.001**
	Yes	6.59	6.28	6.9	
Are most of your friends practicing the social distancing?	No/unknown	5.45	5.19	5.71	**<0.001**
	Yes	6.67	6.26	7.08	
Could people be unintentionally spreading the COVID-19 virus by touching their cell phones?	No/unknown	5.61	5.35	5.88	**<0.001**
	Yes	6.35	5.94	6.76	
Is Pakistan prepared to provide proper care to people affected by COVID-19 epidemic?	No/unknown	6.97	6.57	7.38	**<0.001**
	Yes	5.31	5.06	5.57	
Is COVID-19 not curable in Pakistan?	No/unknown	7.29	6.8	7.78	**<0.001**
	Yes	5.45	5.21	6.69	

* Mean Score for the COVID-19 prevention behavior scale [Range 0–12]. The number of prevention behaviors refer to number of “yes” responses by each study participant on prevention behaviors items listed in Table 2 under the section “Individual behaviors/actions concerning COVID prevention”. Abbreviations: CI, Confidence Interval; LL, Lower Limit; UL, Upper Limit. Note: Bolded font for *p* indicates the significance of differences in mean at *p* < 0.05.

**Table 4:** Negative binomial regression of the COVID-19 prevention behaviors (scale score) *, Depalpur City, Pakistan, 2020.

Demographic Characteristics and HBM Scales	IRR	95% CI		*p*
		LL	UL	
Gender				
Male	0.90	0.72	1.14	0.38
Female (RC)	1.00			
Education				
No Formal Education	0.96	0.59	1.56	0.87
Less than High School	0.83	0.53	1.30	0.42
High School Diploma	0.98	0.60	1.63	0.95
Intermediate	0.90	0.56	1.46	0.67
Graduation	1.04	0.64	1.67	0.89
Post-Graduation (RC)	1.00			
Age				
Under 20	1.07	0.68	1.68	0.76
20–34 years	1.03	0.72	1.48	0.87
35–49 years	0.94	0.70	1.27	0.71
50 years and above (RC)	1.00			
Marital Status				
Single or Separated/Divorced	1.07	0.80	1.44	0.63
Married (RC)	1.00			
Number of Household members				
Less than five	1.50	0.84	2.66	0.17
Five to Seven	1.33	0.77	2.32	0.31
Eight to Ten	1.35	0.74	2.47	0.33
More than Ten (RC)	1.00			
House Ownership				
No	1.15	0.91	1.45	0.24
Yes (RC)	1.00			
Perceived Susceptibility to Disease Scale (3 items)	0.90	0.78	1.05	0.18
Perceived Severity of Disease Scale (3 items)	0.96	0.82	1.12	0.62
Perceived Benefits of Preventative Actions Scale (3 items)	1.16	1.06	1.28	** <0.001 **
Perceived Barriers of Preventative Action Scale (2 items)	1.03	0.87	1.22	0.71
Cues to Action Scale (2 items)	1.02	0.81	1.29	0.85

* The COVID-19 preventive behaviors/actions scale score consisted of a sum of 12 items, each coded as yes = 1 and no/don’t know = 0, with potential ranges from 0 to 12. Abbreviations: IRR, Incidence Rate Ratios; CI, Confidence Interval; LL, Lower Limit; UL, Upper Limit; RC, Reference Category. Note: Bolded font for *p* indicates the significance of differences in mean at *p* < 0.05.

## Data Availability

The data presented in this study are available on request from the corresponding author. The data are not publicly available due to informed consent, assuring the participants that data will not be used for any other purpose other than presenting the findings in a summary form.

## Supplementary Materials

The following are available online at https://www.mdpi.com/article/10.3390/ijerph18136772/s1: Figure S1, Questionnaire in Urdu; Table S1, Comparison of Mean Score for the COVID-19 Prevention Behavior by Socio-Demographic Characteristics, Depalpur City, Pakistan, 2020.
