# Supplementary material for: Perceived Benefits Matter the Most in COVID-19 Preventive Behaviors: Empirical Evidence from Okara District, Pakistan
Source: Int J Environ Res Public Health. 2021 Jun 24;18(13):6772. doi: 10.3390/ijerph18136772 (PMC8297269; doi:10.3390/ijerph18136772)
Supplement: Supplementary file 1 [file ijerph-18-06772-s001.zip › Questionnaire_final.pdf]

Figure S1. Questionnaire

Perceived Benefits Matter the Most in COVID-19 Preventive Behaviors: Empirical Evidence from Okara District City, Pakistan

Objective of Study:

The objective of this study is to get the information on spread of coronavirus (COVID-19) and assess prevention behavior among people from the general population in District Okara, Pakistan. No identifying information about study participants will be released. Only results from this survey will be published, in order to help public health experts and administrators recommend effective measures over the future. You may skip any question you feel uncomfortable answering.

Do we have your consent to participate in this survey?

(a) Yes
(b) No (Stop the participating in the survey)

Demographic Profile

1) **Name** (optional)____________
2) **Which of the following categories represents your age?**
   a) Under 20 **b)** 20-34 years **c)** 35-49 years **d)** 50 years and above
3) **Gender**
   a) Male **b)** Female
4) **What is the name of the city where you currently reside?** _______________
5) **What is your level of education?**
   a) No formal education **b)** less then high school **c)** high school diploma
   d) Intermediate **e)** Graduation **f)** Post graduation
6) **Which of the following best represents your profession/employment status?**
   a) Labourer **b)** Salaried person **c)** Businessman
   d) Unemployed **e)** Other (Please specify)
7) **What is your current marital status?**
   a) Single **b)** Married **c)** Other (e.g. widowed, divorced, and separated)
8) What is the number of family members in your household?
   a) Less than five **b)** five to seven **c)** eight to ten **d)** More than ten
9) **Which of the following best represent the monthly income of your household?**
   a) Less than 30,000 **b)** 30,000 to 60,000 **c)** 60,000-99,000 **d)** More than 100,000

We would like to ask you some questions
About COVID-19 and social distancing.

Sr.no	Behavior and action	Yes	No	Not sure or prefer not to answer
10	Do you live in your own house?			
11	Are you living with joint family system?			
12	Have you heard about COVID-19?			
13	Do you have any medical history?			
14	Have you ever been tested for COVID-19?			
15	Are you aware of the common sign and symptoms of COVID-19?			
16	Do you believe COVID-19 related self-isolation and social distancing affect the human body or human mind?			
17	Do you leave your home during the lockdown?			
18	Do you think that the social distancing slows the rate of COVID-19?			
19	Are you wearing gloves when you go out of your home?			
20	In your Opinion, is your life/family affected negatively by social distancing?			
21	Are you avoiding any non-essential travel?			
22	Are you staying six feet away from the other people?			
23	Are you staying/working at home rather than going to work or school?			
24	Are you self-quarantining if you have the virus or believe you have the virus?			
25	Are you avoiding going to the grocery store or pharmacy?			
26	Are you avoiding all social gatherings (large and small)?			
27	Are you avoiding using public transportation (except essential service workers)?			

**28**	Are you avoiding going for walks in the parks?			
**29**	Are you washing your hands everyday with soap and water for 20 seconds after you go out of your home?			
**30**	Has COVID-19 lockdown helped Pakistan prevent its spread?			
**31**	Are you following basic protective measures (e.g. hand washing, use of mask in public, social distancing) to protect yourself against the COVID-19?			
**32**	Do you think, that schools should resume quickly after the lockdown period with proper emphasis on social distancing, following the Covid-19 pandemic?			
**33**	Do you think going to school, hospitals or any institution is safe for you?			
**34**	Do you think, social distancing is effective in keeping you safe from COVID-19?			
**35**	Are most of your friends practicing the social distancing?			
**36**	Could people be unintentionally spreading the COVID-19 virus by touching their cell phones?			
**37**	Is Pakistan prepared to provide proper care to people affected by COVID-19 epidemic?			
**38**	Is COVID-19 curable in Pakistan?			

<u>Thank you for your valuable time and participation</u>
